# The Delta SARS-CoV-2 Variant of Concern Induces Distinct Pathogenic Patterns of Respiratory Disease in K18-hACE2 Transgenic Mice Compared to the Ancestral Strain from Wuhan

**DOI:** 10.1128/mbio.00683-22

**Published:** 2022-04-14

**Authors:** Xiang Liu, Helen Mostafavi, Wern Hann Ng, Joseph R. Freitas, Nicholas J. C. King, Ali Zaid, Adam Taylor, Suresh Mahalingam

**Affiliations:** a Menzies Health Institute Queensland, Griffith University, Gold Coast, Queensland, Australia; b Global Virus Network (GVN) Centre of Excellence in Arboviruses, Griffith University, Gold Coast, Queensland, Australia; c School of Medical Sciences, Griffith University, Gold Coast, Queensland, Australia; d The Discipline of Pathology and Bosch Institute, School of Medical Sciences, Sydney Medical School, The University of Sydney, Sydney, NSW, Australia; e Marie Bashir Institute for Infectious Diseases and Biosecurity, Sydney Medical School, The University of Sydney, Sydney, NSW, Australia; Johns Hopkins Bloomberg School of Public Health

**Keywords:** Delta variant, SARS-CoV-2, coronavirus, mouse model

## Abstract

Compared to the original ancestral strain of SARS-CoV-2, the Delta variant of concern has shown increased transmissibility and resistance toward COVID-19 vaccines and therapies. However, the pathogenesis of the disease associated with Delta is still not clear. In this study, using K18-hACE2 transgenic mice, we assessed the pathogenicity of the Delta variant by characterizing the immune response following infection. We found that Delta induced the same clinical disease manifestations as the ancestral SARS-CoV-2, but with significant dissemination to multiple tissues, such as brain, intestine, and kidney. Histopathological analysis showed that tissue pathology and cell infiltration in the lungs of Delta-infected mice were the same as in mice infected with the ancestral SARS-CoV-2. Delta infection caused perivascular inflammation in the brain and intestinal wall thinning in K18-hACE2 transgenic mice. Increased cell infiltration in the kidney was observed in both ancestral strain- and Delta-infected mice, with no clear visible tissue damage identified in either group. Interestingly, compared with mice infected with the ancestral strain, the numbers of CD45^+^ cells, T cells, B cells, inflammatory monocytes, and dendritic cells were all significantly lower in the lungs of the Delta-infected mice, although there was no significant difference in the levels of proinflammatory cytokines between the two groups. Our results showed distinct immune response patterns in the lungs of K18-hACE2 mice infected with either the ancestral SARS-CoV-2 or Delta variant of concern, which may help to guide therapeutic interventions for emerging SARS-CoV-2 variants.

## INTRODUCTION

A novel coronavirus causing acute respiratory illness in humans, later named severe acute respiratory syndrome coronavirus 2 (SARS-CoV-2), emerged in Wuhan, the capital and the largest metropolitan area of Hubei province, China, in late 2019 (1). The virus quickly spread around the world, infecting millions of people and causing a global pandemic of coronavirus disease 2019 (COVID-19) (2). A wide range of clinical signs and symptoms, from asymptomatic to severe illness and death, have been reported to be associated with COVID-19. Mild symptoms include fever, cough, fatigue, muscle aches, headache, and sore throat (3). COVID-19 patients with more severe disease can experience dyspnea, pneumonia, and acute respiratory distress syndrome (ARDS) (3, 4). As of 4 February 2022, the World Health Organization (WHO) has reported over 386.5 million confirmed clinical cases of COVID-19 and 5.7 million fatalities cumulatively worldwide (https://covid19.who.int/).

Coronaviruses belong to family *Coronaviridae*, order *Nidovirales*, and realm *Riboviria*. The coronavirus genome comprises a positive-sense, single-stranded RNA between 26 and 32 kb in length, encoding 16 nonstructural proteins and 13 structural proteins. The RNA genome is packed in a nucleocapsid of helical symmetry which is coated with an outer envelope. The envelope is a lipid bilayer anchored with viral structural proteins, including membrane protein, envelope protein, and spike protein, which mediates viral attachment to—and fusion with—host cells by interacting with angiotensin-converting enzyme 2 (ACE2) receptor on target cells (5). The virus-host protein-protein interface, in addition to the high mutation rate of RNA viruses (6), places evolutionary pressures on SARS-CoV-2 to optimize infection and evade host immune responses (7, 8). Multiple SARS-CoV-2 variants were detected in different geographical regions around the world just a few months after the initial Wuhan outbreak (9, 10).

The SARS-CoV-2 Delta variant (Pango lineage B.1.617.2; GISAID clade G/478K.V1) was first detected in clinical samples from India in October 2020. As of December 2021, the Delta variant had spread to over 180 countries and was classified as a variant of concern (VOC) by the WHO. Delta was found to be highly contagious with a basic reproduction number (*R*_0_) of 5.08, nearly 2-fold higher than the ancestral Wuhan-1-Hu strain (*R*_0_ of 2.79) (11). Pfizer and AstraZeneca COVID-19 vaccines developed in the first year of the pandemic were shown to have reduced effectiveness against Delta (10, 12). Compared to the genome sequence of the ancestral strain (GenBank accession no. NC_045512.2), Delta carries 13 amino acid mutations in the spike protein, four of which (L452R, T478K, P681R, and D614G) are of particular concern due to their role in promoting immune escape and infectivity. L452R was reported to enhance SARS-CoV-2 antibody resistance (13, 14) and increase spike protein stability, therefore promoting viral replication (15). The mutation T478K was reported to stabilize the interaction between the spike protein receptor-binding domain (RBD) and ACE2 receptor by increasing the electrostatic potential at the interface (16). The mutation P681R is thought to be another crucial feature of the Delta strain, as this mutation facilitates the cleavage of the spike precursor protein into the active S1/S2 conformation which promotes the release of new virus particles (17). D614G, which is present in subdomain 2 of spike protein, was reported to promote the replication and transmission of the Delta variant in both animal models and humans (18–21).

The disease pathogenesis of Delta infection, including virus dissemination, severity of disease, and the immune response profile, remains unclear. Small-animal models of SARS-CoV-2 infection have provided fundamental insights into the pathogenesis of COVID-19 (22). The K18-hACE2 transgenic mouse model, in which human ACE2 (hACE2) receptor is expressed in mouse airway and epithelial cells, was developed in 2007 for the purpose of investigating SARS-CoV-induced respiratory disease (23). This mouse model is susceptible to SARS-CoV-2 infection and typically manifests as severe COVID-19-like disease symptoms, including lung inflammation and dysfunction (24, 25). In this study, using K18-hACE2 transgenic mice, we describe and analyze the pathogenicity of Delta infection. Observations from this study will provide knowledge on the pathogenesis of a SARS-CoV-2 variant of concern, give insights into the virulence determinants of Delta, and shed light on the development of potential therapeutic approaches in the current COVID-19 global health crisis.

## RESULTS

### Disease and weight loss following SARS-CoV-2 ancestral strain and Delta infection in K18-hACE2 mice.

**FIG 1:**
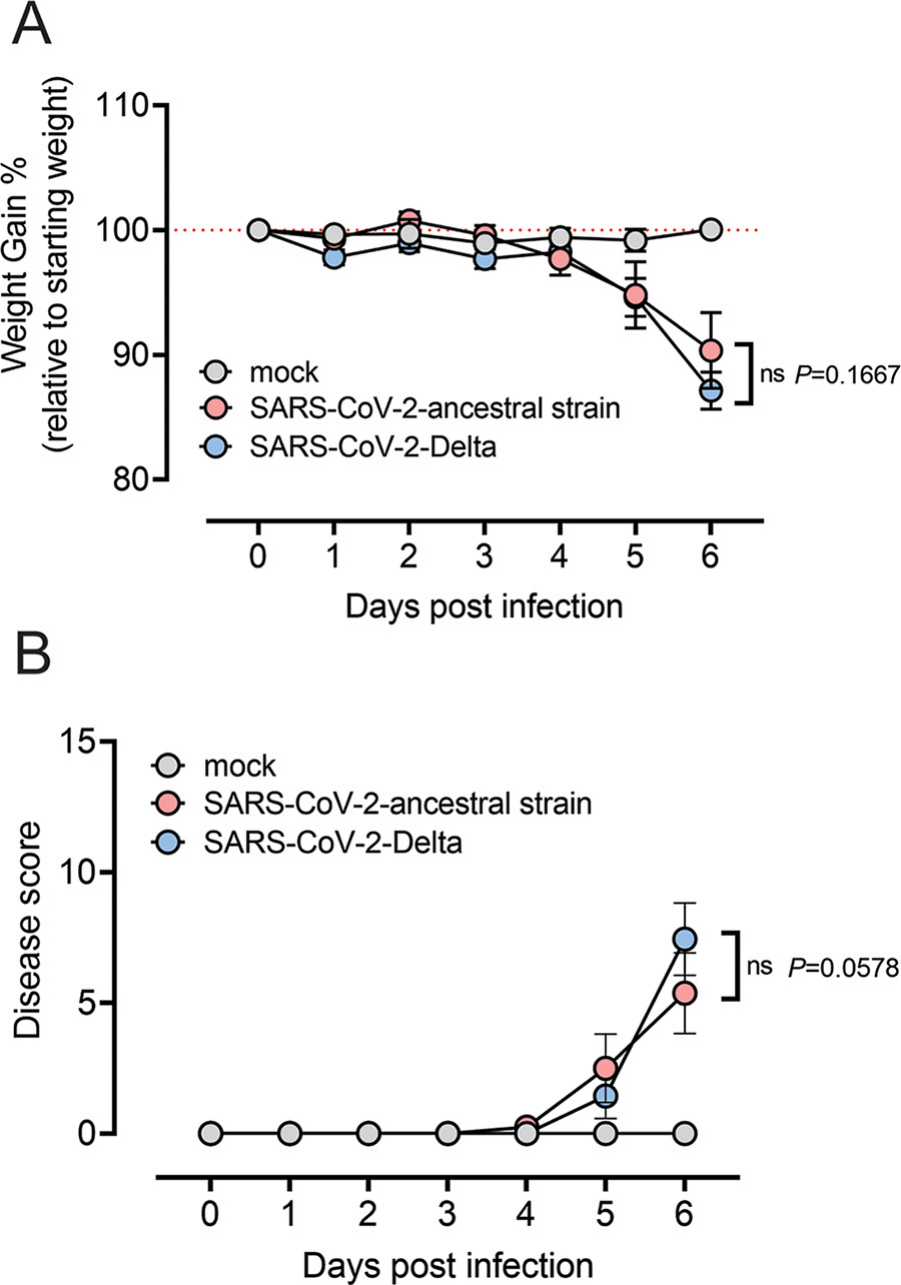
Disease and weight loss of the mice infected by SARS-CoV-2 ancestral strain and Delta variant. Eight-week-old female K18-hACE2 mice were intranasally inoculated with PBS (mock group) or 10^4^ PFU of SARS-CoV-2 ancestral or Delta strain (*n* = 8). Mock-infected mice (*n* = 4) received 20 μL sterile DMEM with 2% FBS intranasally. Individual mice were monitored daily until reaching a clinical score of >3, when twice-daily monitoring was performed. (A) Weight change was monitored and compared with the initial weight on day 0. (B) Mice were given a disease score according to general health (eating habit, locomotion, and behavior), appearance, and weight loss. All values represent the means ± standard errors of the mean from one experiment. Data were analyzed using two-way analysis of variance (ANOVA) with Bonferroni *post hoc* test (ns, not significant).

SARS-CoV-2-infected K18-hACE2 mice have been shown to recapitulate the clinical signs of severe COVID-19, including a decline in pulmonary function, upregulated innate immune responses in the lung, pulmonary inflammation, and respiratory failure, making them an ideal model to further investigate COVID-19 pathogenesis and compare the diseases caused by SARS-CoV-2 variants (25). To assess the disease induced by the SARS-CoV-2 ancestral strain and Delta infection, 8-week-old female K18-hACE2 mice were inoculated intranasally with 10^4^ PFU of SARS-CoV-2 ancestral strain or SARS-CoV-2 Delta strain in a volume of 20 μL. Disease development and weight loss were monitored daily until 6 days postinfection (dpi). At 5 dpi, mice infected with both ancestral and Delta SARS-CoV-2 showed significant weight loss (approximately 6%) (Fig. 1A). Ancestral strain- and Delta-infected mice showed similar disease manifestations (*P* = 0.0578), including body weakness, restricted movement, labored breathing, and dry eyes (Fig. 1B). By 6 dpi, ancestral strain- and Delta-infected mice had lost approximately 10% to 13% of body weight and showed a disease score between 5 and 7.

### Virus replication and tissue tropism of SARS-CoV-2 ancestral strain and Delta in infected K18-hACE2 mice.

**FIG 2:**
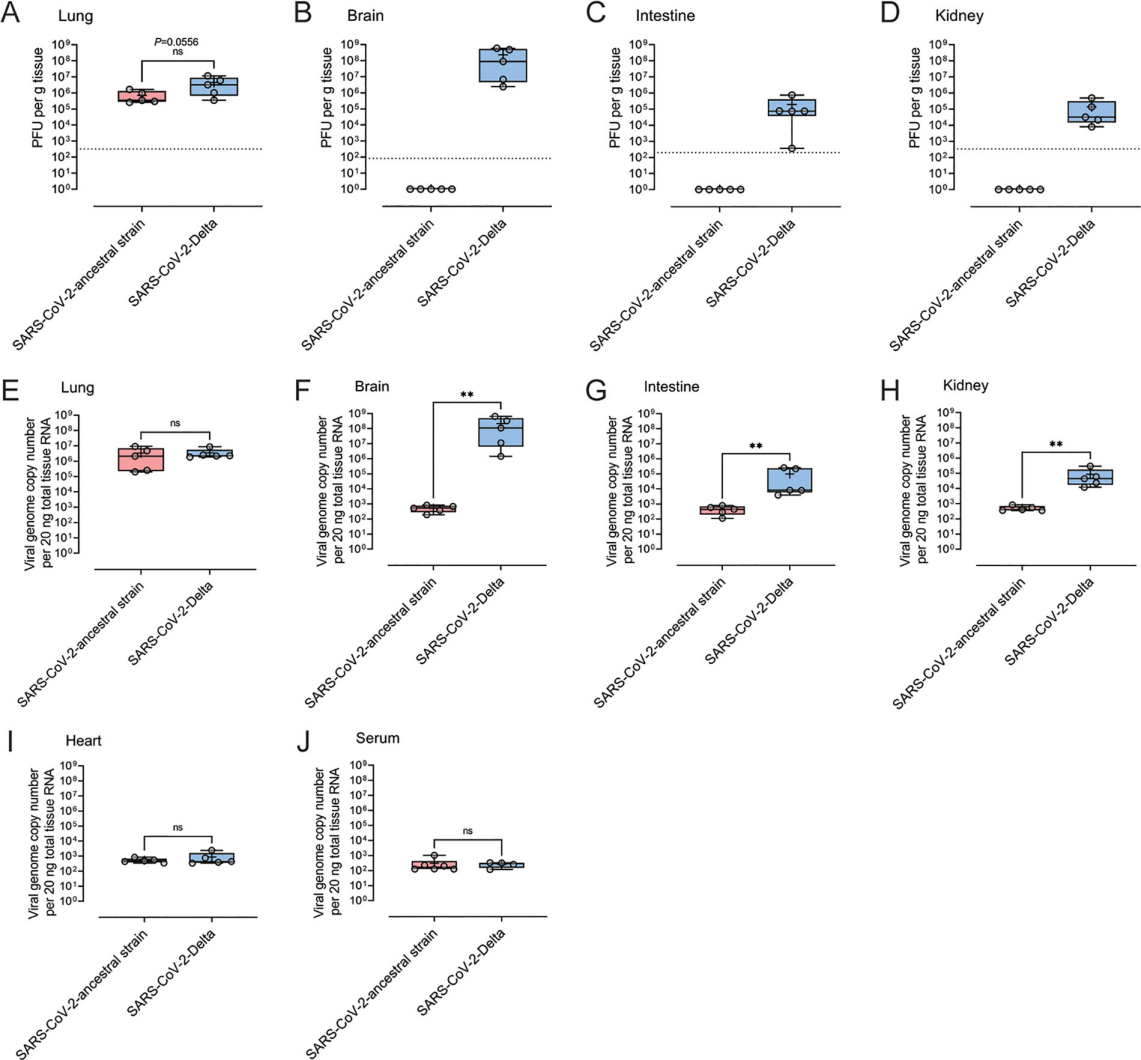
Viral burden in tissues of the mice infected by SARS-CoV-2 ancestral strain and Delta variant. Eight-week-old female K18-hACE2 mice were intranasally inoculated with PBS (mock group) or 10^4^ PFU of SARS-CoV-2 ancestral or Delta strain (*n* = 5). At 6 dpi, the titers of infectious virus in the lung (A), brain (B), intestine (C), and kidney (D) were determined by plaque assay. The copy numbers of viral genome in lung (E), brain (F), intestine (G), kidney (H), heart (I), and serum (J) were determined by probe-based qRT-PCR. Each symbol represents the mean titer for one mouse. Data were analyzed by Mann-Whitney test (**, *P* < 0.01; ns, not significant).

To determine virus load and dissemination of SARS-CoV-2 ancestral strain and Delta in K18-hACE2 mice, infected mice were euthanized, and tissues were dissected for analysis by plaque assay and qualitative real-time PCR (qRT-PCR) at 6 dpi. High levels of infectious virus were detected by plaque assay in the lung tissue of mice infected with ancestral SARS-CoV-2 (∼7 × 10^5^ PFU/g of tissue) and Delta (∼4 × 10^6^ PFU/g of tissue) (*P* = 0.0556) (Fig. 2A). Although the difference was not statistically significant, the higher average viral load in the lung tissue of Delta-infected mice than that of ancestral strain-infected mice was approaching statistical significance (*P* = 0.0556). We detected infectious virus in the brain (∼2.3 × 10^8^ PFU/g tissue), intestine (∼1.9 × 10^5^ PFU/g tissue), and kidney (∼1.4 × 10^5^ PFU/g tissue) of all mice infected with Delta but none of the mice infected with the ancestral strain (Fig. 2B to D). No infectious virus was detected in the serum or heart tissue of mice infected with either the ancestral or Delta strain at 6 dpi (data not shown). The copy number of viral genomes in tissues was quantified by qRT-PCR. There was no significant difference in the viral copy number in the lungs of mice infected with the ancestral or Delta strain (Fig. 2E). However, in brain, intestine, and kidney, the copy number of the Delta genome was significantly higher than that of ancestral strain-infected mice (Fig. 2F to H). The viral genome copy numbers of both ancestral strain and Delta were at similarly low levels in heart tissue and serum (Fig. 2I and J).

### Histopathological analysis of lung, brain, intestine, and kidney tissue following SARS-CoV-2 ancestral strain and Delta infection.

**FIG 3:**
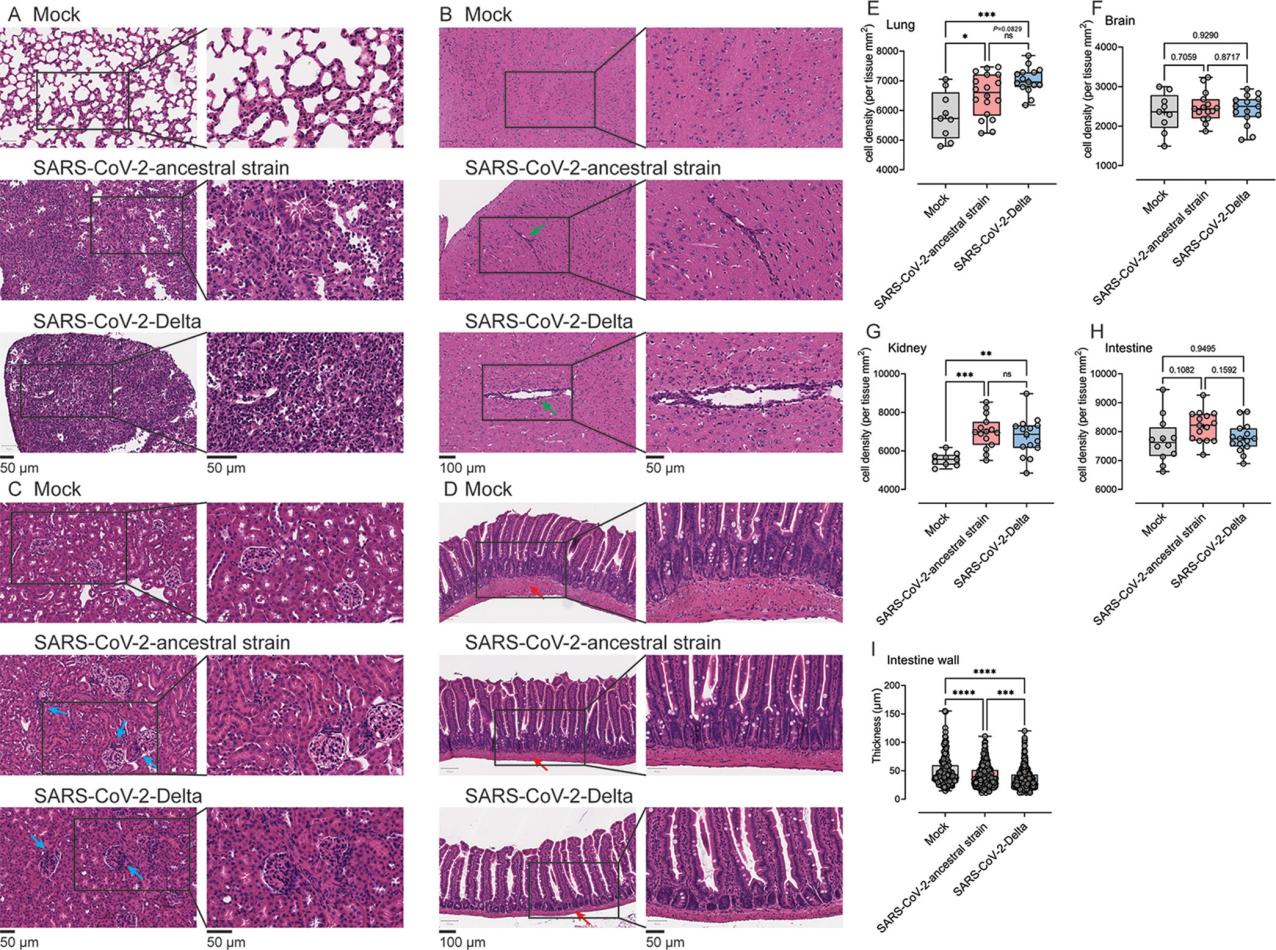
Histopathological analysis of lung, brain, kidney, and intestine from the mice infected by SARS-CoV-2 ancestral strain and Delta variant. Eight-week-old female K18-hACE2 mice were intranasally inoculated with PBS (mock group) or 10^4^ PFU of SARS-CoV-2 ancestral or Delta strain (*n* = 5). At 6 dpi, lung (A and E), brain (B and F), kidney (C and G), and intestine (D, H, and I) tissues were collected and processed with H&E staining. Cell density of the whole-tissue region was quantified using ImageScope. Arrows: green arrows, perivascular infiltration (B); blue arrows, cell infiltration (C); red arrows, intestine wall (D). Each image is representative of ≥3 mice. Statistical analysis performed using one-way ANOVA with Bonferroni *post hoc* test (ns, not significant; *, *P* < 0.05; **, *P* < 0.01; ***, *P* < 0.001; ****, *P* < 0.0001).

To assess the histopathology of the disease associated with the ancestral and Delta strain infection in K18-hACE2 mice, lung, brain, intestine, and kidney of infected mice were collected at 6 dpi. Multiple areas of the lung in ancestral strain- and Delta-infected mice manifested moderate (1/5 in ancestral strain group; 2/5 in Delta group) to severe (4/5 in ancestral strain group; 3/5 in Delta group) pathological changes, typified by prominent immune cell infiltration surrounding blood vessels, thickened interalveolar septa, and consolidation with necrotic debris. Most of the pathological changes were located in the marginal areas of the lung or in proximity to apical or anterior basal regions. No significant difference in the number of cells infiltrating the lungs of ancestral strain- and Delta-infected mice was observed histologically (*P* = 0.0829) (Fig. 3A and E). In the brain, localized vascular congestion, perivascular cuffing, and mononuclear cell infiltration in the cerebral cortex of the brain were observed in both ancestral strain- and Delta-infected mice (Fig. 3B and F), with occasional meningeal or choroidal plexus infiltrates. Notably, this was more prominent in Delta-infected mice (3/5 mice showed perivascular infiltration) than in mice infected with ancestral strain (1/5 mice showed perivascular infiltration), consistent with our observation that live Delta and Delta viral RNA were detected in the brain at higher levels than ancestral strain. In the kidney, no clear pathology due to SARS-CoV-2 infection was identified by histological analysis (Fig. 3C). However, occasional enhanced glomerular infiltration by mononuclear cells was observed in both ancestral strain- and Delta-infected mice (Fig. 3G). In the small intestine of infected mice, no morphological changes were observed in the villi of the small intestine or any increase in general cellular infiltration (Fig. 3D and H), although occasional small focal basal mononuclear cell infiltrates were noted in Delta-infected mice. However, the intestinal wall, which is composed of circular and longitudinal layers of smooth muscle, was significantly thinner in Delta-infected than in mock- and ancestral strain-infected mice (Fig. 3I).

### Inflammatory gene expression in the lungs of SARS-CoV-2 ancestral strain- and Delta-infected K18-hACE2 mice.

**FIG 4:**
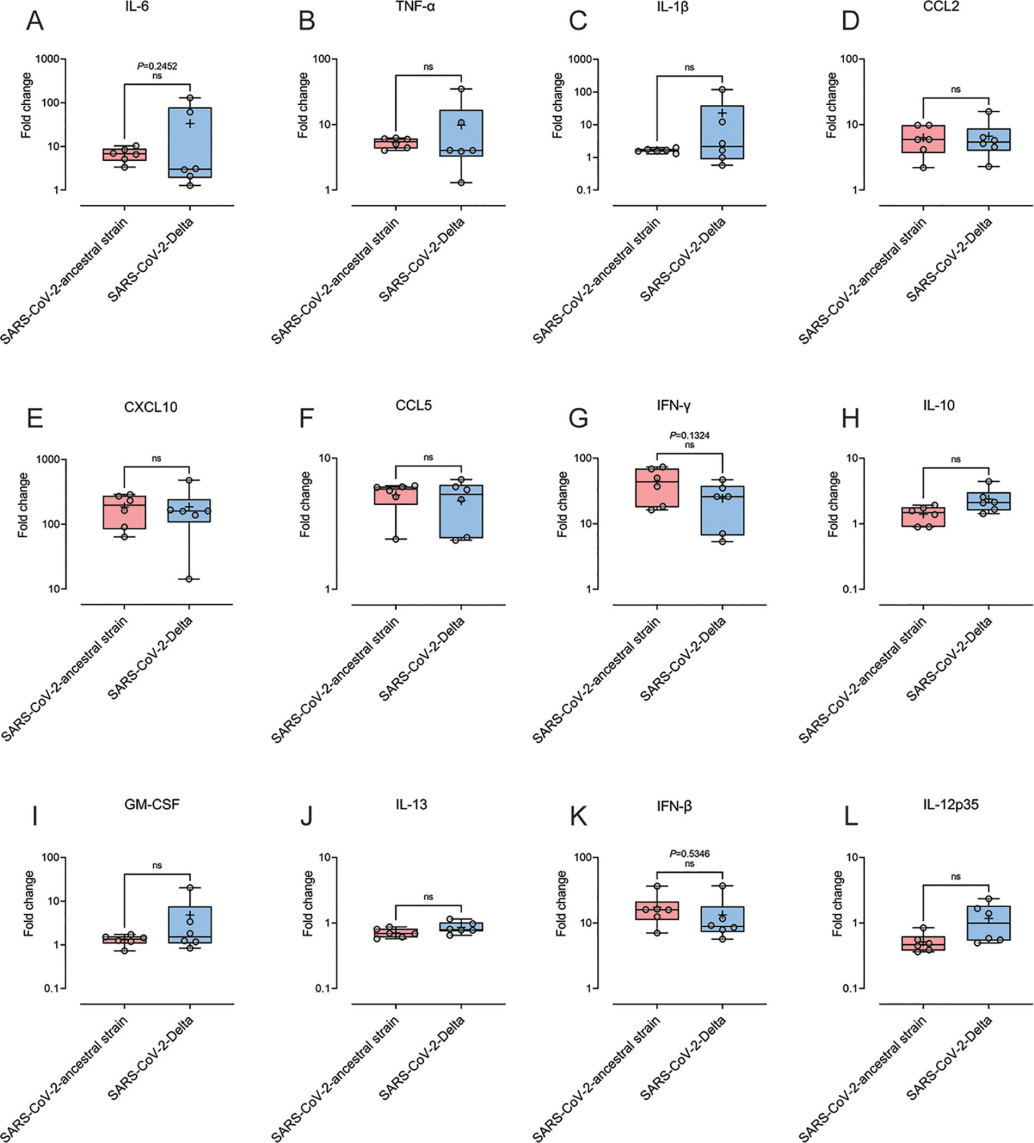
Cytokine and chemokine profile in the lung tissue of the mice infected by SARS-CoV-2 ancestral strain and Delta variant. Eight-week old female K18-hACE2 mice were intranasally inoculated with PBS (mock group) or 10^4^ PFU of SARS-CoV-2 ancestral or Delta strain (*n* = 6). Transcriptional profiles of immune mediators, IL-6 (A), TNF-α (B), IL-1β (C), CCL2 (D), CXCL10 (E), CCL5 (F), IFN-γ (G), IL-10 (H), GM-CSF (I), IL-13 (J), IFN-β (K) and IL-12p35 (L), were determined by qRT-PCR in the lung at day 6 post infection. Data were normalized to HTRP levels and are shown as fold change from the level in mock-infected mice. Each symbol represents the mean titer for one mouse. Data were analyzed by Student's *t* test (ns, not significant).

Cytokine storm, characterized by excessive production of proinflammatory cytokines such as interleukin 6 (IL-6), tumor necrosis factor alpha (TNF-α), and IL-1β, is reported to contribute to ARDS in patients with severe COVID-19 (26, 27). We therefore measured the transcription levels of proinflammatory cytokines and chemokines in the lung tissue of the mice infected with SARS-CoV-2 ancestral strain or Delta at 6 dpi. Compared to mock-infected mice, the expression of IL-6, TNF-α, IL-1β, CCL2, CXCL10, CCL5, gamma interferon (IFN-γ), IL-10, granulocyte-macrophage colony-stimulating factor (GM-CSF), IL-13, IFN-β, and IL-12p35, in both infected groups, was all upregulated in lung homogenates (Fig. 4). No significant differences were identified in any of these factors between ancestral strain- and Delta-infected groups.

### Leukocyte profile of the lungs of SARS-CoV-2 ancestral strain- and Delta-infected K18-hACE2 mice.

**FIG 5:**
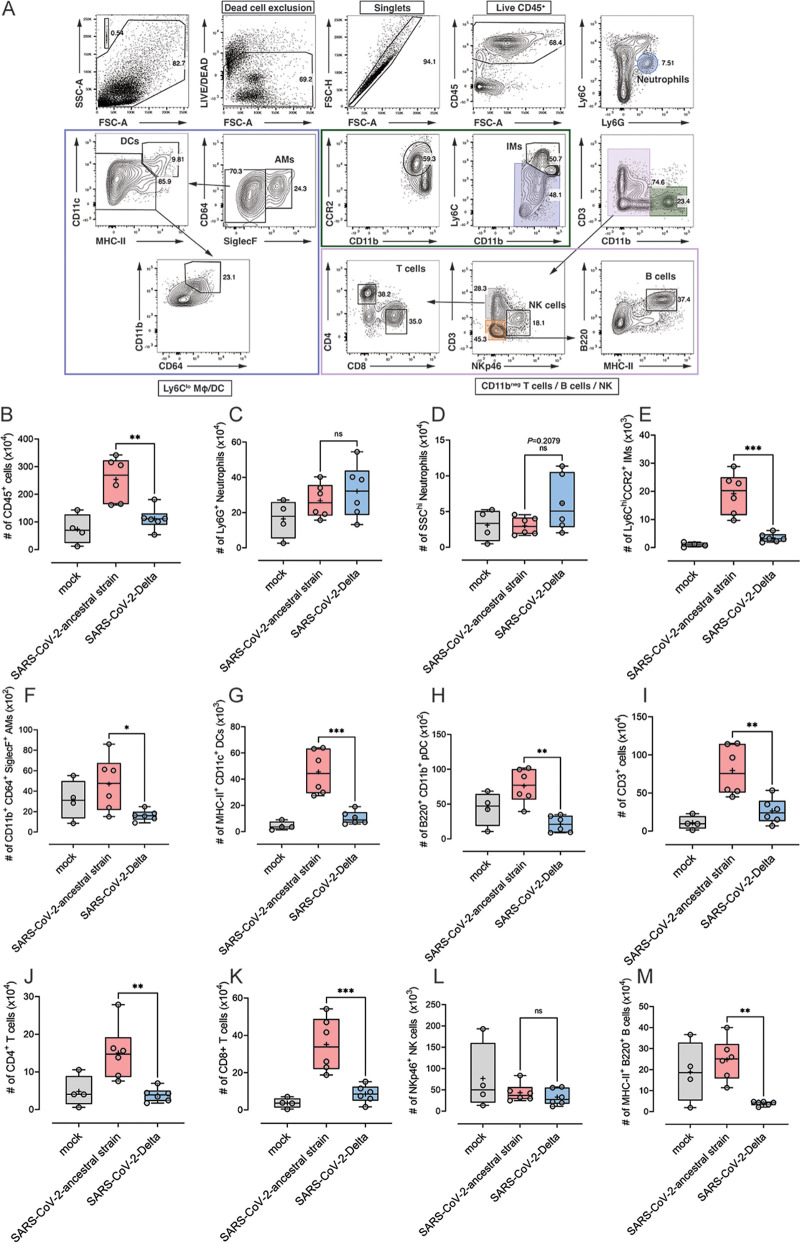
Leukocyte profile in the lung tissue of the mice infected by SARS-CoV-2 ancestral strain and Delta variant. Eight-week-old female K18-hACE2 mice were intranasally inoculated with PBS (mock group) or 10^4^ PFU of SARS-CoV-2 ancestral or Delta strain (*n* = 4 for mock group; *n* = 6 for infection groups). At 6 dpi, lung tissues were collected and processed for flow cytometry. Gating strategy for lung leukocytes (A), the numbers of CD45^+^ leukocytes (B), Ly6G^+^ neutrophils (C), SSC^hi^ neutrophils (D), Ly6C^hi^ CCR2^+^ inflammatory monocytes (IM) (E), CD11b^+^ CD64^+^ SiglecF^+^ alveolar macrophages (F), MHC-II^+^ CD11c^+^ dendritic cells (DCs) (G), B220^+^ CD11b^+^ plasmacytoid dendritic cells (pDCs) (H), CD3^+^ T cells (I), CD4^+^ T cells (J), CD8^+^ T cells (K), NK cells (L), and MHC-II^+^ B220^+^ B cells (M) were analyzed. Data were analyzed by one-way ANOVA with Bonferroni *post hoc* test (ns, not significant; *, *P* < 0.05; **, *P* < 0.01; ***, *P* < 0.001).

Immune cell dysregulation induced by SARS-CoV-2 infection has been suggested to contribute to pulmonary pathology in COVID-19 patients (28). To assess the immune cell response in mice infected with SARS-CoV-2 ancestral strain or Delta, lung tissue was analyzed by flow cytometry at 6 dpi (Fig. 5A). Interestingly, the numbers of CD45^+^ immune cells were significantly higher in the lungs of mice infected with ancestral strain, compared to mice infected with Delta (Fig. 5B). Moreover, the numbers of inflammatory monocytes (IM), alveolar macrophages (AM), dendritic cells, and CD4^+^ and CD8^+^ T cells and B cells were all found to be significantly higher in ancestral strain-infected mice than in mice infected with Delta (Fig. 5E to K and M). No significant difference was found in the numbers of NK cells and Ly6G^+^ neutrophils between the two infected groups (Fig. 5C and L). Delta-infected mice had slightly more SSC^hi^ neutrophils in lung tissue than mice infected with the ancestral strain; however, this was not statistically significant (Fig. 5D).

### Immunofluorescence analysis of lung tissue from SARS-CoV-2 ancestral strain- and Delta-infected K18-hACE2 mice.

**FIG 6:**
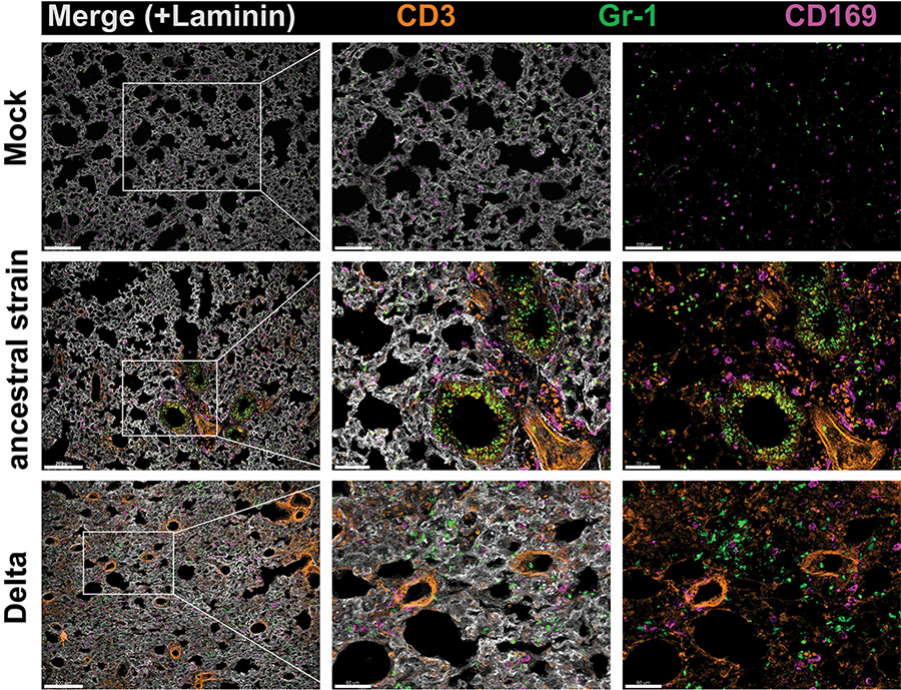
Immunofluorescence analysis of the lung tissue from K18-hACE2 mice infected by SARS-CoV-2 ancestral strain and Delta variant. Eight-week-old female K18-hACE2 mice were intranasally inoculated with PBS (mock group) or 10^4^ PFU of SARS-CoV-2 ancestral or Delta strain (*n* = 4 for mock group; *n* = 6 for infection groups). At 6 dpi, lung tissues were collected and processed for immunofluorescence staining. Lung cryosections were labeled with CD3 (T cells), Gr-1 (neutrophils), CD169 (alveolar macrophages), and laminin (parenchymal tissue). Colocalization and distribution of CD3^+^ T cells, Gr-1^+^ neutrophils, and CD169^+^ alveolar macrophages are shown in the image. Images representative of *n* = 6 mice per group were acquired by confocal microscopy as a z-stack using a 10× (1.0-NA) objective. Scale bars in panels = 150 mm.

To further characterize the leukocyte profile in infected lung tissue, immunofluorescence analysis of T cells, neutrophils, and CD169^+^ alveolar macrophages was performed. Compared to the lung tissue of mock-infected mice, higher levels of CD3^+^ T cells were present in the parenchyma of ancestral strain- and Delta-infected mice. T cells were mainly distributed in the adventitia around bronchioles (Fig. 6). Gr-1^+^ neutrophils in both ancestral strain- and Delta-infected mice were sparsely distributed in the lung parenchyma; however, neutrophils in the lungs of Delta-infected mice were more localized in proximity to bronchiole mucosal fold areas (Fig. 6). In the lungs of both ancestral strain- and Delta-infected mice, CD169^+^ alveolar macrophages were sparingly distributed in the lung tissue, with no significant difference in the distribution observed between the two infected groups (Fig. 6).

## DISCUSSION

Transmissibility, vaccine breakthrough, and disease severity are the three biggest concerns for human health when characterizing new emerging SARS-CoV-2 variants. The Delta variant of concern has been shown to be more transmissible (29), and vaccine effectiveness against Delta is reduced, compared to the ancestral strain (30, 31). However, there is very limited information on the disease severity associated with Delta infection. There are a number of factors that make it difficult to compare the disease profile of Delta infection to that of the ancestral strain. For example, the Delta variant originated from different geographic regions than did the ancestral strain of SARS-CoV-2, making it prevalent in different patient populations with different genetic backgrounds and access to diverse health care resources. Additionally, as the COVID-19 pandemic has progressed, patients infected with Delta have been treated under different clinical guidelines compared to those at the start of the pandemic. A meta-analysis based on 26 studies of disease severity and clinical outcomes showed that infection with Delta increases the risk of hospitalization by 2.08-fold (95% confidence interval [CI], 1.77 to 2.39), the risk of intensive care unit (ICU) admission by 3.35-fold (95% CI, 2.5 to 4.2), and the risk of mortality by 2.33-fold (95% CI, 1.45 to 3.21), compared to the ancestral strain (32). Notably, the analysis shows that Delta has the highest risk of ICU admission and mortality compared to the Alpha, Beta, and Gamma variants. A recent study to determine pathogenicity of the Delta variant conducted by Lee et al. showed a higher disease score in K18-hACE2 mice infected with Delta compared with the Alpha variant (hCoV19/England/204820464/2020, GISAID: EPI_ISL_683466) (33). We observed comparable disease severity and weight loss over 6 days in K18-hACE2 mice infected with ancestral or Delta SARS-CoV-2. Our observations are similar to a recent study in which Syrian hamsters infected with ancestral or Delta strain (at 10^5^ PFU per animal) developed comparable levels of weight loss from 5 dpi and both peaked at 7 dpi (34). Because the hamster model of SARS-CoV-2 infection is a nonlethal model, infected hamsters recovered quickly after peak weight loss (34). Additional kinetic investigations based on more time points and bigger sample size would provide further details of disease development in K18-hACE2 mice.

The disease observed in ancestral strain- and Delta-infected mice was associated with high viral load in lung tissue. Importantly, in K18-hACE2 mice, Delta infection spread beyond pulmonary tissues to various organs, including brain, kidney, and intestine, where live virus and viral RNA were detected. The viral RNA level in the brain of Delta-infected mice was ∼10^5^-fold higher than that of ancestral strain-infected mice. However, our observations differ from the study by Lee et al. that showed low levels (below detection limit) of Delta viral RNA in mouse brain at 6 dpi (33). Further studies on the brain tropism of Delta are therefore recommended. Interestingly, compared to the difference in brain viral RNA, the difference in viral RNA load in the intestines and kidney between ancestral strain- and Delta-infected mice was only approximately 100-fold. The expression level of ACE2 in organs is likely to affect virus tissue tropism, as Delta’s enhanced ability to disseminate in K18-hACE2 mice may be due to the enhanced entry efficiency of its spike protein on recognizing the hACE2 receptor (17, 35). However, the brain and kidney of K18-hACE2 mice express comparable levels of hACE2 (25, 36). The mechanism of tissue tropism of Delta warrants further investigation. It is also worth noting that, compared to the recently emerged Omicron variant (B.1.1.529), the Delta strain showed no difference in the viral load in nasal turbinates but more profound infection in lung tissue of infected hamsters (34).

Based on a meta-analysis involving 241 COVID-19 patients, diffuse alveolar damage (DAD), pulmonary hemorrhage and thromboembolism represented common lung-associated pathology (65.1%) (37). Histopathological analysis of ancestral strain- and Delta-infected lung tissue revealed moderate to severe alveolar damage and inflammation in the parenchyma. Similar pathology has been observed in small-animal models (33, 38). The brain has been shown to be another important organ involved in COVID-19 pathology in both clinical patients (37) and K18-hACE2 mice following infection with SARS-CoV-2 clinical isolate USA-WA1/2020 (39–41). SARS-CoV-2-induced lesions in the mouse brain were mainly found in the cerebral cortex. Pathological change in the cerebral cortex may be due to the proximity of the cerebral cortex to the olfactory bulb, as SARS-CoV-2 has been shown to invade the central nervous system through the neural-mucosal interface in olfactory mucosa (42). In line with previous reports, multiple sites of mononuclear cell infiltration were identified within the cerebral cortex region of the brain in both ancestral strain- and Delta-infected mice. More abundant tissue involvement was found in brains from Delta-infected mice than in those from ancestral strain-infected mice, suggesting—at least in part—that, as more infectious virus and viral RNA were found in the brain of Delta-infected than of ancestral strain-infected mice, SARS-CoV-2 infection of brain tissue may have a role in brain pathology in K18-hACE2 mice. Although live ancestral SARS-CoV-2 was not detected at 6 dpi in the brain tissue, the lesions identified in the brain of ancestral strain-infected mice indicate that virus may be replicating at very low levels or being cleared earlier than the Delta strain. Previous studies have identified live virus in the brain of K18-hACE2 mice infected with the ancestral strain at similar disease time points as those analyzed here (43). Further assays to determine virus growth and histopathology of brain tissue at earlier and later time points would help clarify if virus replication correlates with brain pathology. Infectious virus in the brain of the Delta-infected mice did not cause overt cell infiltration at day 6 postinfection. We observed only localized vascular congestion, perivascular cuffing, and mononuclear cell infiltration sparsely distributed in the brain of 3 out of 5 Delta-infected mice and in 1 out of 5 ancestral strain-infected mice. From previous reports, based on either COVID-19 clinical patient samples (44, 45) or animal data (46, 47), microhemorrhages, perivascular neutrophilic/lymphocytic infiltrates, and fibrin microthrombi were detected at various levels in the brain. However, there was no evidence of overall increased cell infiltration in the whole-brain tissue, whether viral RNA genome was present in the tissue or not. Specifically, Song et al. (47) observed that, in the brain tissue of deceased COVID-19 patients, regions with positive viral staining did not show lymphocyte or leukocyte infiltration. Their results suggested that SARS-CoV-2 may not induce immune responses typical of other neurotropic viruses, which induce large numbers of immune cell infiltrates neighboring sites of virus infection (47). Brain damage in COVID-19 patients may result in long-term cognitive sequelae (48). It is unclear whether SARS-CoV-2 replication and associated pathology in the mouse brain will lead to any long-term consequence in mouse behavior. The brain tissue damage observed in this study may help to establish a disease model for future studies. Leukocyte infiltration and tissue damage in the kidneys due to SARS-CoV-2 infection, such as renal tubular necrosis or hemorrhage, have been reported in postmortem analyses (37), multiple small-animal models infected with SARS-CoV-2 Canada/ON/VIDO-01/2020 or a mouse-adapted strain, MASCp36 (49, 50), and in clinical studies (51, 52). In our study, glomerular mononuclear cell infiltration was observed to similar degrees in ancestral strain- and Delta-infected mice, but no clear tissue pathology was identified in the kidney. Further study is needed to elucidate the link between virus replication and cell infiltration in kidney tissue. SARS-CoV-2 has been shown to infect intestinal tissue and cause leukocyte infiltration and morphological changes in small-animal models infected with SARS-CoV-2 Canada/ON/VIDO-01/2020 or USA-WA1/2020 (36, 49) and in humans (53, 54). In this study, thinning of the intestine wall in response to SARS-CoV-2 infection was observed. The intestine wall in Delta-infected mice was found to be thinner than that in mock- and ancestral strain-infected mice, which again may be a result of higher levels of infectious Delta and Delta RNA genome present in the intestinal tissue. Thinning of the small intestine wall is thought to be associated with intestinal pneumatosis (55), which has been reported in COVID-19 patients (56). Thinning of the intestine wall may also be attributed to excessive gas in the intestine of infected mice (57) or the worsening nutritional status of SARS-CoV-2-infected mice. SARS-CoV-2 Canada/ON/VIDO-01/2020 and USA-WA1/2020, SARS-CoV-2 clinical isolates used in previous animal studies (36, 39, 40, 49), have only three amino acid mutations (ORF1a [I1607V], E [S6L], and N [S194L]) and one mutation (L84S in ORF8), respectively, compared to the ancestral strain. The two isolates were therefore both genetically close to the original ancestral strain (Nextstrain). Thus, our comparison here of the tissue damage following ancestral strain and Delta infection is valuable in determining the difference in pathogenesis of COVID-19 caused by emerging SARS-CoV-2 variants. Future studies on the histopathology of other COVID-19-related organs, such as heart, liver, spleen, colon, and reproductive organs, would help to further decipher SARS-CoV-2 variant-induced pathology.

In agreement with previous clinical reports showing cytokine storm is correlated with severe COVID-19 (3), elevated levels of proinflammatory cytokines were detected in the lung tissue of ancestral strain- and Delta-infected mice. However, there was no significant difference in cytokine expression between the two infected groups. The comparable levels of cytokine profile we observed in ancestral strain- and Delta-infected mice concur with the similar disease manifestations of the two groups, as cytokine storm has been shown to correlate directly with COVID-19 severity (27). The study by Lee et al. detected higher levels of proinflammatory cytokines (TNF-α, IL-1β, CXCL13, IL-6, and CXCL10) which were associated with more severe disease in Delta-infected K18-hACE2 mice, compared to the Alpha variant (hCoV19/England/204820464/2020; GISAID: EPI_ISL_683466) (33). Future studies on cytokine/chemokine profile at both the protein level (e.g., multiplex Luminex assay) and the transcriptional level (e.g., RNA sequencing and NanoString) would provide a more comprehensive outlook on the role of cytokines in COVID-19.

Immature/total neutrophils were reported to be increased in severe COVID-19 patients (58). In our study, although not statistically significant, both the clinical disease score and the number of infiltrating SSC^hi^ neutrophils in Delta-infected mice were higher than in mice infected with the ancestral strain, which is in line with the observation made in the work of Carissimo et al. (58). Moreover, IL-6 also appeared to be higher in Delta-infected mice than in mice infected with the ancestral strain, although not statistically significantly, supporting the correlation between immature neutrophil counts and IL-6 (58). As neutrophil-derived extracellular traps (NETs) have been reported to be induced by SARS-CoV-2 infection and associated with lung tissue damage (lung epithelial apoptosis) in COVID-19 patients (59), it would be interesting to investigate whether NETs contribute to the higher disease (*P*_disease_ = 0.0578) and cell infiltration (*P*_lung cell infiltration_ = 0.0829) in the lungs of Delta-infected K18-hACE2 mice. The numbers of CD45^+^ cells, T cells, B cells, inflammatory monocytes, and dendritic cells (DCs) in the inflamed lung tissue of Delta-infected mice were significantly lower compared to mice infected with the ancestral strain. This was despite disease manifestations, lung virus titers, and proinflammatory cytokines/chemokines being at comparable levels between the two groups. Although leukocytes have been shown to be recruited into lung tissue following SARS-CoV-2 infection in K18-hACE2 mice (25) and COVID-19 patients (60), various reports regarding the correlation between total leukocytes and COVID-19 severity have been published (61). Several studies have shown that surviving patients usually present with lower total leukocyte counts, compared to nonsurviving patients (62). However, leukopenia has also been reported in COVID-19 patients in several studies (3, 63). A clinical report based on 326 COVID-19 patients with pneumonia at normal, low, or high levels suggested that leukocyte numbers, at least in the blood, do not closely corelate with COVID-19 severity (64, 65). Furthermore, a clinical study of 31 COVID-19 patients showed that the percentage of infection in the lung was negatively correlated with peripheral blood lymphocyte percentage and count but positively correlated with neutrophil percentage in the blood (66). In our study, the disease severity, virus replication, and pathology in lungs of Delta-infected mice were all slightly higher (close to significant) than with the ancestral strain, although the differences were not statistically significant (*P*_disease_ = 0.0578; *P*_lung virus titer_ = 0.0556; *P*_lung cell infiltrates_ = 0.0829). Furthermore, the levels of IFN-γ and IFN-β both trended lower in Delta-infected mice, compared to that of ancestral strain-infected mice (*P*_IFN-γ_ = 0.1324; *P*_IFN-β_ = 0.5346). The low levels of leukocytes in the lungs observed in Delta strain-infected mice may therefore be due to as yet unknown immune evasion mechanisms. Related to this, the mechanism behind the differences in leukocyte recruitment between ancestral strain and Delta infections in mice warrants further investigation, such as exploring the determinants of disease severity by investigating the role of essential leukocytes (T cells, monocytes, alveolar macrophages, etc.) in the pathogenesis of COVID-19. Notwithstanding these differences, the severe histopathological changes in the lungs of both ancestral strain- and Delta-infected animals are consistent with the progressive compromise of pulmonary function, rather than the functional failure of other organs.

In summary, using a K18-hACE2 mouse model, the Delta variant disseminated to multiple tissues and caused more tissue damage than the ancestral strain of SARS-CoV-2. Numbers of leukocytes recruited to the lung tissue during Delta infection were lower than with the ancestral strain, suggesting Delta may have a novel invasion and evasion mechanism by which the host leukocyte response was suppressed, facilitating dissemination. As new SARS-CoV-2 variants, such as Omicron, continue to emerge, knowledge of the variant-specific pathogenesis of variants of concern will be valuable for the optimization and development of therapeutic strategies.

## MATERIALS AND METHODS

### Animal ethics statement.

Animal experiments were approved by the Animal Ethics Committee of Griffith University (protocol no. MHIQ/07/20/AEC). All procedures conformed to the Australian Code for the Care and Use of Animals for Scientific Purposes (67).

### Viruses.

SARS-CoV-2-ancestral strain (GenBank accession no. NC_045512.2, MN908947.3) was generated as previously described (43, 68). Briefly, 10 μg of pCC1-4K-SARSCoV-2-Wuhan-Hu1 (GenBank accession no. MT926410) was transfected into BHK-21 cells using Lipofectamine LTX with Plus reagent (Life Technologies) according to the manufacturer’s instructions. At 24 h posttransfection, the BHK-21 cells were resuspended and the whole-cell slurry was transferred onto a monolayer of Vero E6 cells. Cell supernatant was collected as virus P0 stock at 48 to 72 h posttransference or until 50% cytopathic effect (CPE) was observed. The SARS-CoV-2 Delta (Pango lineage B.1.617.2; GISAID clade G/478K.V1) strain (VIC18440) was a kind gift from Nigel A. J. McMillan. This strain was originally obtained from the Peter Doherty Institute for Infection and Immunity and Melbourne Health, Victoria, Australia.

### Mouse infections and disease monitoring.

K18-hACE2 C57BL/6 mice were obtained from the Animal Resource Centre (Perth, Australia). Eight-week-old female K18-hACE2 mice were anesthetized with ketamine-xylazine and inoculated intranasally with 10^4^ PFU of SARS-CoV-2 ancestral strain or SARS-CoV-2 Delta strain in a volume of 20 μL. Mock-infected mice received 20 μL of sterile Dulbecco’s modified Eagle’s medium (DMEM) with 2% fetal bovine serum (FBS). The mice were monitored for disease manifestations daily. Mice were scored using a cumulative and progressive clinical disease matrix. Mice were given a score between 0 and 3 for each of the following health indicators: eating habit, locomotion, behavior and appearance, and weight loss, as well as general health (including breathing, body weakness, eye symptoms, etc.). A score of 0 was normal. After receiving an overall score of >3, mice were monitored twice daily. A score of 3 in any one of the 5 categories (e.g., >15% weight loss) or an overall score of >10 led to the mouse being euthanized.

### Plaque assay.

The titers of infectious virus were enumerated by plaque assay (69). Mouse tissues were collected in 1× phosphate-buffered saline (PBS) and homogenized using the Bead Ruptor 24 Elite homogenizer according to the manufacturer’s instructions. The supernatant was collected by centrifugation at 12,000 × *g*, 4°C, for 10 min and stored at −80°C for longer preservation. Vero E6 cells were seeded in 12-well plates at 2.5 × 10^5^ cells per well and cultured in DMEM with 5% FBS overnight. The cells were infected with a dilution series of virus from samples and overlaid with 1.2% colloidal microcrystalline cellulose (Sigma-Aldrich) in DMEM supplemented with 2% FBS. The plates were incubated for 72 h at 37°C. Cells were fixed with 4% paraformaldehyde (PFA) and stained with 0.1% crystal violet. Viral titters were calculated using the following formula: PFU/mL = (average number of plaques/volume [mL] of virus added) × the dilution factor.

### Flow cytometry.

The infected mice were culled and perfused with PBS. Lung tissues were collected for flow cytometry (fluorescence-activated cell sorting [FACS]) analysis (70). Lungs were minced and digested in type IV collagenase (0.2 mg/mL; Worthington) with DNase I (0.05 mg/mL; Sigma) and passed through 70-μm cell strainers. Cells were stained with antibodies for 45 min in FACS buffer (PBS with 0.5% [wt/vol] bovine serum albumin [BSA] and 2 mM EDTA). Fluorochrome-conjugated monoclonal antibodies against mouse CD45 (30-F11), CD3 (17A2), CD8 (53.6-7), CD4 (RM.4-5), Ly6C (HK1.4), CD11b (M1/70), Ly6G (1A8), B220 (RA3-6B2), CD11c (N418), SiglecF (E50-2440), NKp46 (29A1.4), major histocompatibility complex class II (MHC-II) (M5/114.15.2), CD64 (454-5/7.1), CCR2 (475301), and CD106 [429(MVCAM.A)] (all purchased from eBiosciences) were used, and near-infrared (NIR) LIVE/DEAD stain (Thermo Fisher) was used to exclude dead cells. Counting beads (Spherobeads; BD) were added to the samples before acquisition. Cell populations were analyzed on a BD LSRFortessa cell analyzer with BD FACSDiva software, version 6.1.3. Data analysis was performed with FlowJo (TreeStar, Inc.) software, version 9.0.

### Immunofluorescent histology and confocal microscopy.

Lung tissues were harvested and fixed in 4% paraformaldehyde overnight. The tissues were subsequently washed in PBS and dehydrated in in 30% sucrose overnight. The tissues were embedded in optimal-cutting-temperature (OCT) compound (Sakura Finetek) and then frozen at −80°C. Tissue blocks were cut into 20-μm-thick sections and permeabilized in cold acetone. The sections were blocked with serum-free protein block (Dako) and immunolabeled with antibodies against mouse CD3 (2A7; BioLegend), Gr-1 (RB6-8C5; BD Biosciences), CD169 (3D6-1.12; AbD Serotec), and laminin (rabbit polyclonal; Abcam). The slides were mounted with ProLong Gold antifade agent (Thermo Fisher). Images were acquired using a confocal microscope (Olympus FV3000) using a 10× and 20× (0.95-numerical-aperture [NA]) objective and processed using Imaris (v9.5; Bitplane).

### Qualitative real-time PCR assay for mouse cytokines.

The infected mice were culled and perfused with PBS. Mouse tissues of interest were collected in TRIzol (Invitrogen) and homogenized using the Bead Ruptor 24 Elite homogenizer according to manufacturer’s instructions. Total RNA was extracted from tissue homogenates using TRIzol (Invitrogen) according to the manufacturer’s instructions. RNA was reverse transcribed into cDNA using reverse transcriptase (Sigma-Aldrich) with random primers. SYBR green real-time PCR was performed on a CFX96 Touch real-time PCR system (Bio-Rad) to measure the expression of inflammatory mediators. Primers for HTRP (housekeeping gene), IL-6, TNF-α, IL-1-β, CCL2, CXCL10, CCL5, IFN-γ, IL-10, GM-CSF, IL-13, IFN-β, and IL-12p35 were purchased from Qiagen. SYBR green real-time PCR conditions were set as follows: (i) 1 cycle of 95°C for 15 min and (ii) 40 cycles of 94°C for 15 s, followed by 55°C for 30 s and 72°C for 30 s. The DNA amplification specificity was evaluated by melting curve analysis. The mRNA level of each target gene was expressed as fold change from the level in mock-infected samples.

### Qualitative real-time PCR (qRT-PCR) assay for viral genomic RNA.

The infected mice were culled and perfused with PBS. Lung, heart, brain, kidney, intestine, and serum were collected in TRIzol reagent (Invitrogen) and homogenized using the Bead Ruptor 24 Elite homogenizer according to manufacturer’s instructions. Total RNA was extracted and reverse transcribed into cDNA using reverse transcriptase (Sigma-Aldrich) with random primers. QuantiTect probe real-time PCR was performed on a CFX96 Touch real-time PCR system (Bio-Rad) to measure the viral genome copy numbers. SARS-CoV-2 RdRp gene-targeting primer and probe sequences were 5′-GTGAAATGGTCATGTGTGGCGG-3′ (forward), 5′-CAAATGTTAAAAACACTATTAGCATA-3′ (reverse), and 6-carboxyfluorescein (FAM)–CAGGTGGAACCTCATCAGGAGATGC-black hole quencher (BHQ) (probe sequence). The QuantiTect probe real-time PCR conditions were set as follows: (i) 1 cycle of 95°C for 15 min and (ii) 40 cycles of 94°C for 15 s followed by 60°C for 1 min. The DNA amplification specificity was evaluated by melting curve analysis. Viral genome copy number was calculated against a standard curve by a viral infectious clone (GenBank accession no. MT926410).

### H&E staining.

The infected mice were culled and perfused with PBS. Lung, kidney, brain, and intestine tissues were collected and fixed in 4% PFA. Paraffin blocks of the tissues were cut into 5-μm-thick sections and stained with hematoxylin and eosin (H&E). Images of the tissues were taken by an Aperio AT2 digital whole-slide scanner (Leica). Tissue cell density was quantified using ImageScope software (Algorithm Nuclear v9) using the threshold settings: image zoom of 0.5, a minimum nuclear size (μm^2^) of 10, a maximum nuclear size (μm^2^) of 100, a minimum roundness of 0.4, a minimum compactness of 0.4, and a minimum elongation of 0.2.

### Statistical analysis.

Data for mouse disease scores and weight loss were analyzed using two-way analysis of variance (ANOVA) with a Bonferroni *post hoc* test. Flow cytometry results and cell density results were analyzed using one-way analysis of variance (ANOVA) with a Bonferroni *post hoc* test. Plaque assays and viral genome copy number data were analyzed using the Mann-Whitney test. Cytokine RNA qRT-PCR data were analyzed using Student’s *t* test. All data (except for mouse disease scores) were tested for normality using the D’Agostino-Pearson normality test prior to analysis. All statistical analyses were performed with GraphPad Prism software version 9.0.
